# 730 Clinical utility of the portable pressure measuring device for garment therapy on burn scar

**DOI:** 10.1093/jbcr/irac012.284

**Published:** 2022-03-23

**Authors:** Seung Yeol Lee, Hyun Seok

**Affiliations:** Soonchunhyang University Hospital, Bucheon-si, Kyonggi-do; Soonchunhyang University Hospital, Bucheon, Kyonggi-do

## Abstract

**Introduction:**

The experimenters developed a portable pressure measuring device using silicon piezoresistive pressure sensors. The purpose of this study was to determine the effectiveness of pressure garment therapy using proposed device with objective data obtained through a randomized controlled trial.

**Methods:**

Pressure measurements were acquired through a readout circuit consisting of an analog-to-digital converter, a microprocessor, and a Bluetooth transmission module for wireless data transmission to an external device. This was a double-blinded, randomized, controlled trial in patients with hypertrophic scars. In the pressure monitoring group, garment pressures were monitored using the portable pressure measuring device, and the compression garment was adjusted so that the pressure was maintained at the therapeutic range of 15 – 25 mmHg. In the control group, non-surgical standard treatment of burn scars except for pressure monitoring was performed in the same manner.

**Results:**

No significant intergroup difference was noted at the initial evaluations (p >0.05) between two groups. The pre- to post-treatment change in the scar thickness (p=0.03) and erythema (p=0.03), more reductions were found in the pressure monitoring group than control group. There were no significant differences in the change measurements between the two groups for melanin levels (p=0.62) and transepidermal water loss (TEWL) (p=0.94). The changes (skin distensibility, biological skin elasticity, gross skin elasticity, and skin viscoelasticity) measured with the cutometer showed no significant differences between the two groups (p=0.87, p=0.32, p=0.37, and p=0.29, respectively).

**Conclusions:**

Complementary characteristics such as wireless transmission to an external device may allow burn patients to continuously wear the device for real-time measurements. A Portable pressure monitoring device is effective for significantly improving burn-associated scar characteristics.

